# Focal adhesion kinase is involved in the migration of human osteosarcoma cells

**DOI:** 10.3892/ol.2020.12396

**Published:** 2020-12-18

**Authors:** Sitan Feng, Xin Shi, Ke Ren, Sujia Wu, Xiaoliang Sun

Oncol Lett 9: 2670-2674, 2015; DOI: 10.3892/ol.2015.3131

Subsequently to the publication of the above article, an interested reader drew to the authors' attention that, on p. 2672, in Fig. 1A the data panels for the A1/0 h and A2/0 h experiments were strikingly similar.

After having re-examined their data, the authors realized that the A2 osteosarcoma 143B subclone cell line image at 0 h had erroneously been selected to also represent the A1/0 h experiment during the process of figure compilation. The revised version of Fig. 1, showing the corrected data panel for the A1/0 h experiment, is shown on the next page. Note that the inadvertent error made during the process of compiling this figure affected neither the results nor the conclusions reported in this paper, and all the authors agree to this Corrigendum. The authors thank the Editor of *Oncology Letters* for presenting them with the opportunity to publish this Corrigendum, and apologize to the Editor and to the readership of the Journal for any inconvenience caused.

## Figures and Tables

**Figure 1.**
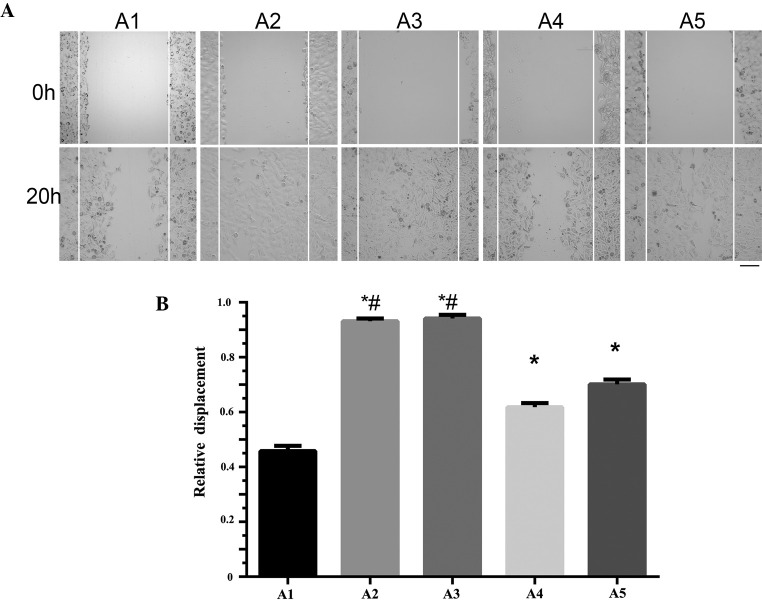
Separation of the osteosarcoma (OS) 143B cell lines with different migration abilities. (A) A wound healing assay was performed on the OS 143B subclone cell lines, A1, A2, A3, A4 and A5. (B) The relative displacement of the osteosarcoma 143B subclone cell lines, A1, A2, A3, A4 and A5. *P<0.05 vs. A1. ^#^P>0.05, A3 vs. A2.

